# Decreased Corneal Endothelial Cell Apoptosis Due to U/S Power Injury With Limbal Mesenchymal Stem Cell Secretome Therapy

**DOI:** 10.1155/sci/2390093

**Published:** 2026-03-02

**Authors:** Dicky Hermawan, I. Ketut Sudiana, Fedik Abdul Rantam, Evelyn Komaratih

**Affiliations:** ^1^ Doctoral Program of Medical Science, Faculty of Medicine, Universitas Airlangga, Surabaya, East Java, Indonesia, unair.ac.id; ^2^ Stem Cell Research and Development Center, Universitas Airlangga, Surabaya, East Java, Indonesia, unair.ac.id; ^3^ Department of Ophthalmology, Faculty of Medicine, Universitas Airlangga, Surabaya, East Java, Indonesia, unair.ac.id; ^4^ Department of Anatomical Pathology, Faculty of Medicine, Universitas Airlangga, Surabaya, East Java, Indonesia, unair.ac.id; ^5^ Faculty of Veterinary Medicine, Stem Cell Research and Development Center, Universitas Airlangga, Surabaya, East Java, Indonesia, unair.ac.id

**Keywords:** apoptosis, corneal endothelial, limbal mesenchymal stem cell, phacoemulsification, U/S power

## Abstract

**Purpose:**

To evaluate the effects of limbal mesenchymal stem cell secretome (LMSC‐S) on corneal endothelial NF‐κB, TNF‐α, and Caspase‐8 expression after U/S power injury of the phacoemulsification machine.

**Setting:**

Stem Cell Research and Development Center, Universitas Airlangga.

**Design:**

Experimental studies on laboratory animals.

**Methods:**

The normal group included eight eyes of four rabbits, and the other groups each included seven eyes of seven rabbits. The normal group consisted of eyes without exposure or treatment. Control group 1 (C1) served as the control for treatment group 1 (T1), where LMSC‐S was administered simultaneously with the U/S power exposure. Control group 2 (C2) served as the control for treatment group 2 (T2), in which LMSC‐S was administered 3 days after U/S power exposure. Corneal endothelial cell (CEC) damage was induced by exposure to ultrasound from a phacoemulsification machine. Rabbit LMSC‐S cells were obtained from the Stem Cell Research and Development Center, Universitas Airlangga. Expression of NF‐κB, TNF‐α, and Caspase‐8 were assessed by immunohistochemistry (IHC).

**Results:**

All studied cytokines increased after U/S power injury (NF‐κB: *p* = 0.047 for N‐C1; *p* < 0.001 for N‐C2, TNF‐α: *p* < 0.001 for N‐C1 and N‐C2, Caspase‐8: *p* < 0.001 for N‐C1 and N‐C2). T2 group showed the least increase and was closer to normal (NF‐κB: *p* = 0.002 for N‐T1, *p* = 0.081 for N‐T2; TNF‐α: *p* = 0.005 for N‐T1, *p* = 0.161 for N‐T2; Caspase‐8: *p* = 0.013 for N‐T1, *p* = 0.739 for N‐T2).

**Conclusions:**

LMSC‐S therapy on the third day postexposure decreased corneal endothelial apoptotic cytokine expression.

## 1. Introduction

Mesenchymal stem cells (MSCs) have emerged primarily as cell‐based therapies for various diseases. Bone marrow MSCs (BMMSCs) were discovered in 1968 by Friedenstein et al. [1]. MSCs have a seemingly ubiquitous localization. They have also been isolated from other adult tissues such as adipose tissue [2], skin [3], lung [4], synovial membranes [5], dental pulp [6], nasal olfactory mucosa [7], breast milk [8], scalp tissue [9], muscle [10], periosteum [11], peripheral blood, endometrial and menstrual blood [12], cervix [13], neonatal/fetal tissue [14–17], and corneal limbus [18, 19]. Although MSCs are present in many tissues, their total number in the body is limited [19–21]. Cell therapy protocols typically require hundreds of millions of MSCs per treatment; therefore, in vitro cell expansion is required for approximately 10 weeks before implantation. Patient age and clinical characteristics influence the optimal culture conditions for the clinical‐scale production of human MSCs [19–21]. Several studies have suggested that the MSC implantation time is too short to achieve an effective effect [22–26]. It has been reported that less than 1% of MSCs survive for more than 1 week after systemic administration [27–31], suggesting that paracrine mechanisms may mediate the primary effects of MSCs [32]. Recent studies have also brought attention to the diverse bioactive factors produced by MSCs, which may play important roles in regulating various physiological processes [33]. Therefore, the secretion of MSCs has attracted much attention due to its potential use in tissue repair and regeneration [19, 32, 34–36].

The corneal endothelium is crucial for maintaining corneal clarity by regulating corneal hydration through the function of corneal endothelial cells (CECs) [37]. Phacoemulsification is the most commonly performed technique in cataract surgery [38]. However, this procedure can damage CECs. As human CECs do not regenerate, wound healing involves enlarging the remaining cells to cover the defective area [39, 40]. Extensive damage can lead to corneal decompensation and bullous keratopathy (BK) [39]. Cataract surgery is the leading cause of BK, accounting for 44.4% of cases. Among them, 39.5%, 22%, and 16.8% were attributed to phacoemulsification, extracapsular cataract extraction (ECCE), and intracapsular cataract extraction (ICCE), respectively [38]. Corneal endothelial dysfunction therapy is corneal transplantation, but this therapy has two main problems: the lack of corneal donors and a decrease in CEC density that occurs during storage, the transplantation process, and within 5 years after transplantation [1, 2]. Alternative therapies are needed to replace or reduce corneal transplantation, which is highly dependent on corneal donors. In experimental studies, apoptosis was significantly induced in CECs during phacoemulsification [41–43].

Damaged CECs due to U/S power injury will release damage‐associated molecular patterns (DAMPs) [44–46]. DAMPs will bind to Toll‐like receptors (TLRs) from inflammatory cells (macrophages), which will activate NF‐κB. Activated NF‐κB will translocate to the cell nucleus and induce DNA synthesis of pro‐inflammatory cytokines such as TNF‐α, IL‐1, IL‐6, and IL‐8 [45, 46]. NF‐κB regulates TNF‐α expression. In contrast, the autocrine–paracrine loop of TNF‐α induces NF‐κB transcription factor expression [47]. TNF, together with its Fas ligand (FasL), can be specifically recognized by the appropriate death receptors, such as Fas or TNF receptor (TNFR)‐1, which are located on the plasma membrane. Their binding will activate the death receptors. Fas can bind Fas‐associated death domain (FADD) or TNFR‐associated death domain (TRADD) and cause FADD aggregation and the appearance of death effector domain (DED). This exposed DED interacts with the DED in the prodomain of proCaspase‐8, inducing the oligomerization of proCaspase‐8, which is localized on the cytosolic side of the plasma membrane. This then forms a large complex known as the death‐inducing signal complex (DISC). Within the DISC, the two linear subunits of proCaspase‐8 interact, leading to the autoactivation of proCaspase‐8 into Caspase‐8. Caspase‐8 can also activate mitochondrial‐mediated pathways through Bid (a member of the pro‐apoptotic Bcl‐2 family) by cleaving it into its active form (tBid). tBid triggers mitochondrial pathways, such as cytochrome c, apoptosis‐inducing factor (AIF), and other factors. They will be released from mitochondria, and apoptosis will be induced. After cytochrome c is released from mitochondria to the cytosol, it activates proCaspase‐6 through Caspase‐9, resulting in the formation of Caspase‐6. Caspase‐6 is the only cytosolic caspase with the ability to activate proCaspase‐8. In the cytochrome c‐dependent pathway, activation of proCaspase‐8 does not require interaction with FADD or DISC [48]. This study tried to see apoptosis from the inflammatory cytokine Caspase‐8, which can be activated through two pathways: the binding of TNF‐α with TNFR‐1 due to activation of NF‐κB by (DAMPS), and by Caspase‐6 due to activation of proCaspase‐8 by mitochondrial cytochromec released into the cytosol.

The secretome of MSCs, which contains soluble proteins, such as cytokines and growth factors, offers a potential solution for tissue regeneration therapy. The secretome of MSCs exhibits anti‐inflammatory, pro‐inflammatory, antiangiogenic, and antifibrotic effects [19]. MSCs can be sourced from the limbus, adipose tissue, and bone marrow, and limbal MSC secretome (LMSC‐S) is particularly promising for addressing phacoemulsification‐induced CEC damage [49–51]. This study aimed to evaluate the effect of LMSC‐S administration on reducing corneal endothelial apoptosis by modulating the expression of NF‐κB, TNF‐α, and Caspase‐8 cytokines after ultrasonic power (U/S) injury from a phacoemulsification machine.

## 2. Methods

This study used 36 eyes from 32 New Zealand white rabbits. The experimental protocol was approved by the Faculty of Veterinary Medicine, Universitas Airlangga (Code of Ethics Number 2. KEH.122.09.2022). In this study, there were five groups. The normal group included eight eyes from four rabbits, and each of the groups (Control 1, Treatment 1, Control 2, and Treatment 2) included seven eyes from seven rabbits. The study utilized a true experimental posttest‐only design with a control group. The inclusion criteria were male New Zealand white rabbits aged 16–18 months, weighing between 2.5 and 3.5 kg. This study used male rabbits to avoid bias from sex hormones, such as testosterone or estrogen, on inflammatory responses [52]. The exclusion criteria included rabbits with unhealthy eye conditions, diseases, or the potential to transmit diseases during the study, as determined by veterinarians. Rabbits were excluded from the study if they became sick, died, or developed complications such as scleral perforation, vitreous prolapse, bleeding, or uveitis during or after U/S power exposure.

### 2.1. Endothelial Injury Model

The corneal endothelial damage model was established in the right eye using an aseptic surgical procedure by the same surgeon (Dicky Hermawan), with modifications to the phacoemulsification machine parameters as described by Nemet et al. [53], Rouhbakhshzaeri et al. [54], Kunzman et al. [55], and Ungricht et al. [56]. The eyelids were disinfected with 10% povidone‐iodine solution. The surgical field was narrowed using a sterile cloth, and an eye speculum was installed. The eyeball was disinfected with 5% povidone‐iodine, then irrigated with balanced salt solution (BSS). A corneal incision was made in the superonasal area using a 2.75 mm keratome. The phacoemulsification probe was carefully inserted into the anterior chamber through a corneal incision to avoid damage to the eye structures, especially the cornea and lens. U/S power exposure of the corneal endothelium was performed using a NIDEK CV9000‐R peristaltic phacoemulsification machine with the following parameters: U/S power 70%, panel 10 s on/off, aspiration flow rate (AFR) 50 mL/min, continuous irrigation, bottle height 100 cm, and vacuum 80 mmHg, with a total treatment time of 10 min. The phacoemulsification probe was positioned bevel‐up just below the iris surface without touching the corneal endothelium or lens in the central area of the anterior chamber with a single operator [50, 51, 53].

### 2.2. Treatment With Limbal Mesenchymal Cell Secretome

LMSC‐S from New Zealand white rabbits were obtained from the Stem Cell Research and Development Center, Airlangga University, which was carried out specifically for this study. The isolation, culture, characterization, and secretome production procedures have been described by Hermawan et al. [57]. Intracameral LMSC‐S was administered to the treatment group by injecting 0.2 mL into the anterior chamber using a 1 mL syringe with a 30G needle through a clear corneal puncture in the T1 and T2 groups. In the C1 and C2 groups, 0.2 mL of intracameral BSS injection was administered in the same manner and at the same time as the intracameral LMSC‐S injection in the T1 and T2 groups [50, 51]. In groups C1 and T1, the intracameral injection procedure was performed at the final stage of U/S power exposure after corneal hydration. In groups C2 and T2, the intracameral injection was given on the third day after U/S power exposure.

### 2.3. Measurement of Corneal Endothelium NF‐κB, TNF‐α, and Caspase‐8 Expressions

Immunohistochemistry (IHC) was used to assess NF‐κB, TNF‐α, and Caspase‐8 expression. The IHC kits used in this study were rabbit NF‐κB p65 polyclonal antibody HRP peroxidase‐conjugated BIOSS Antibodies (bs‐0465R‐HRP), rabbit TNF‐α polyclonal antibody BIOSS antibodies (bs‐2081R), and rabbit monoclonal anti‐Caspase‐8 antibody (AA 411‐482) MEDIKBIO MDAB00010. This process began with deparaffinization of the paraffin block slide, followed by soaking in xylol four times, each for 3–5 min. Then continued rehydrating the preparation by soaking in absolute ethanol three times, each for 1–3 min, followed by soaking in 70% ethanol twice, each for 1–3 min. The slide was washed with aqua bidest three times, and the edges were cleaned with dry paper. Then 3% H_2_O_2_ was added, and the mixture was incubated at room temperature for 10 min. Next, the slide was washed three times with phosphate‐buffered saline (PBS), and the edges were cleaned with tissue. Then, 0.025% trypsin was dropped, incubated at 37°C for 6 min, washed with PBS three times, and the edges of the slide were cleaned using dry paper. Then, the Ultra V Block was dropped and incubated at room temperature for 5 min. Clean the edges of the slide; the slide did not need to be washed. Then the diluted (1:100) polyclonal antibodies for NF‐κB and TNF‐α, and the monoclonal antibody for Caspase‐8 were dripped onto the slides and incubated at room temperature for 25–30 min. Then, the slide was washed with PBS three times, and the edges were cleaned with tissue. Next, Biotinylated was dripped onto the surface and incubated at room temperature for 10 min. It was then washed three times with PBS, and the slide edges were cleaned with dry paper. Then, HRP polymer was dripped and incubated at room temperature for 10 min. The slide was washed with PBS three times, and the edges were cleaned with dry paper. Then, DAB chromogen (20 µL/1 mL substrate) was added, and the mixture was incubated at room temperature for 5–15 min in a dark room. Then washed with aqua bidest three times and cleaned. Next, Meyer hematoxylin was stained and incubated at room temperature for 6–15 min. Then the slide was washed with running water three times, and the final wash involved soaking in water for 10 min. Finally, the slide was dried. The slide was covered with a glass cover. The expression of the studied variable (brown color) in cells was observed using a light microscope at 400x magnification across 10 fields of view.

**Figure 1:**
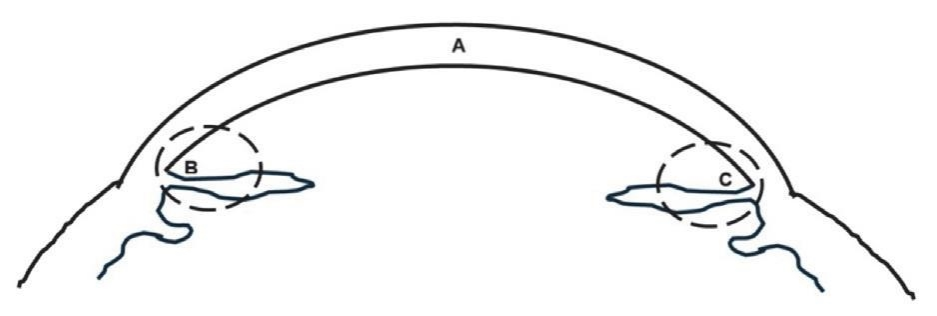
IHC examination areas: A, corneal endothelium; B and C, iris area near the anterior chamber angle.

IHC examination for NF‐κB and TNF‐α expression in macrophages was performed in Areas B and C, and Caspase‐8 expression in CECs was performed in Area A (Figure 1). Macrophages expressing NF‐κB and TNF‐α were observed in 10 fields of view, with five fields each in Areas B and C, while CECs expressing Caspase‐8 were observed in Area A (Figure 1). The average percentage of macrophages expressing NF‐κB/TNF‐α and CECs expressing Caspase‐8 was obtained from the number of positive macrophages divided by the total number of macrophages observed in 10 fields of view.

### 2.4. Termination of Experimental Animals

The New Zealand white rabbits used in this study were euthanized using the decapitation method. Group N was terminated on day zero. Groups C1 and T1 were terminated on the third day after U/S power exposure, and Groups C2 and T2 were terminated on the sixth day.

### 2.5. Statistical Analysis

The collected data were analyzed using SPSS Statistics version 27 (SPSS Inc., Chicago, IL, USA). The data obtained were analyzed in the following order: descriptive statistics to assess the mean, standard deviation, median, minimum, and maximum values, normality test of variable distribution using the Shapiro–Wilk test, homogeneity test of data variation using the Levene test, to test the mean difference between groups using analysis of variance (ANOVA) if the data were normally distributed, then continued with the Bonferroni test if the data were homogeneous or the Brown–Forsythe test followed by the Games–Howell test if the data were not homogeneous; if the data were not normally distributed, using the Kruskal–Wallis test, followed by the Mann–Whitney test. The test was carried out at the 95% confidence level.

## 3. Results

### 3.1. IHC

Figure 2 shows positive NF‐κB, TNF‐α, and Caspase‐8 expression in the cytoplasm of brown‐stained cells. NF‐κB and TNF‐α expression were seen in macrophages in the iris, while Caspase‐8 expression was seen in the CECs (Figure 2).

Figure 2Immunohistochemistry evaluation results in the normal group (N), control 1 (C1), therapy 1 (T1), control 2 (C2), and therapy 2 (T2). Expression of NF‐κB cytokines (A–E), TNF‐α cytokines (F–J), and Caspase‐8 cytokines (K–O). Thick arrows indicate positive cells and thin arrows indicate negative cells. Examination using a light microscope (400x magnification).

**Figure:**
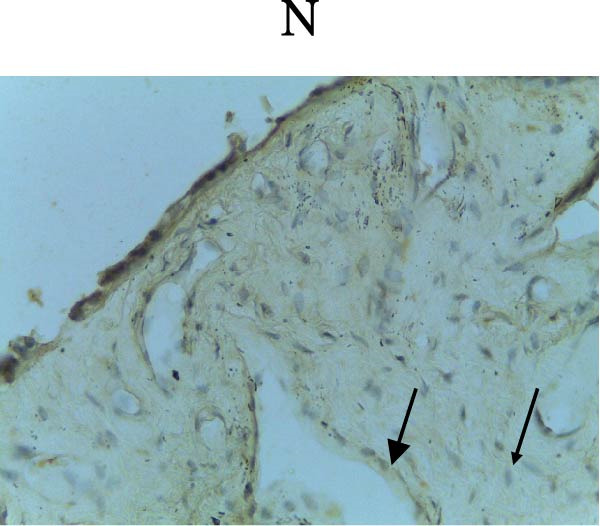
(A)

**Figure:**
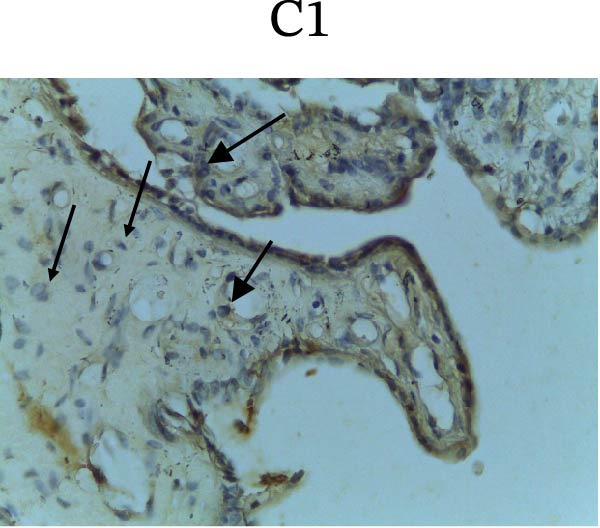
(B)

**Figure:**
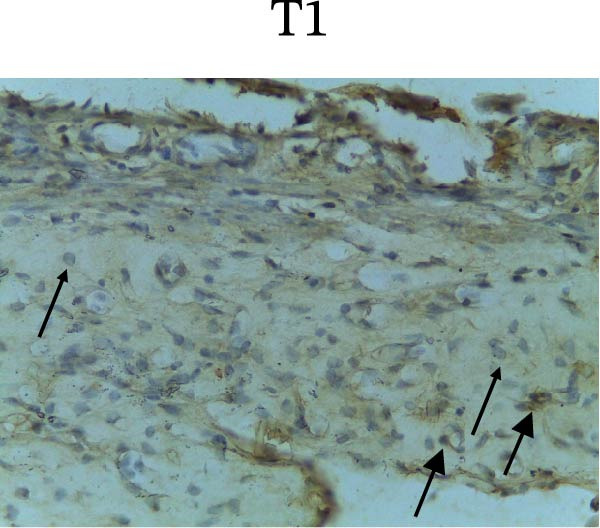
(C)

**Figure:**
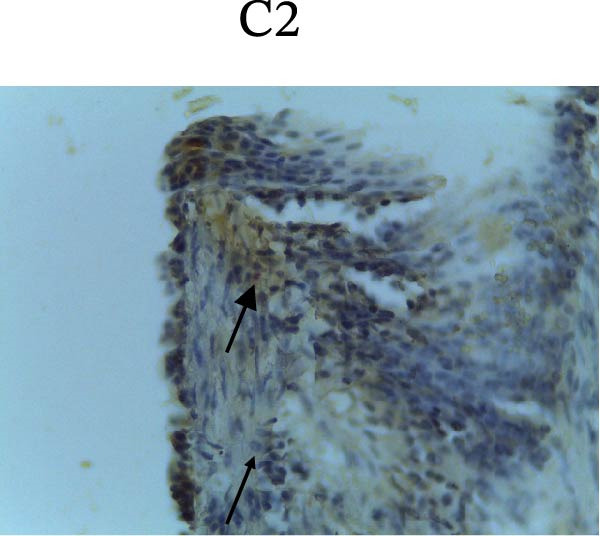
(D)

**Figure:**
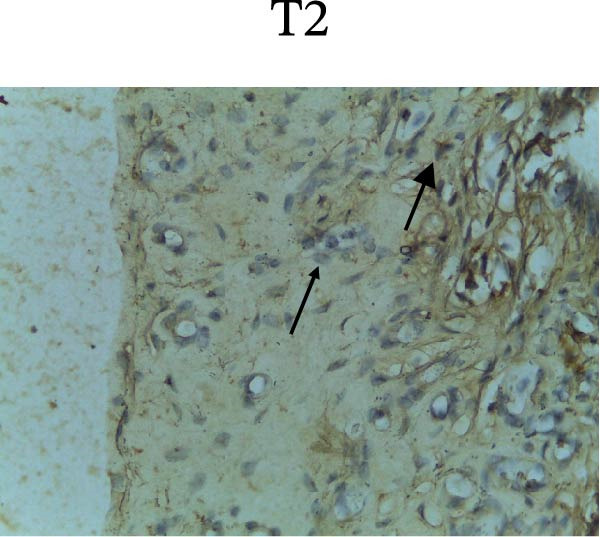
(E)

**Figure:**
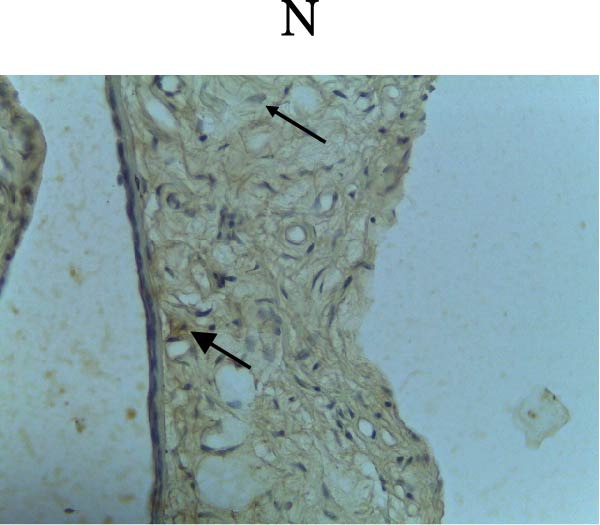
(F)

**Figure:**
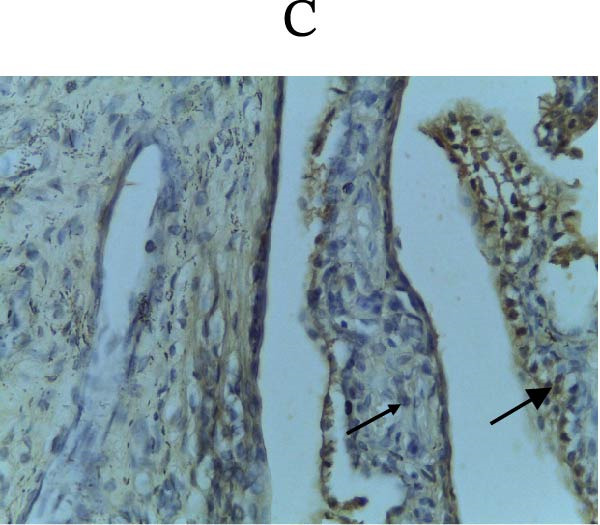
(G)

**Figure:**
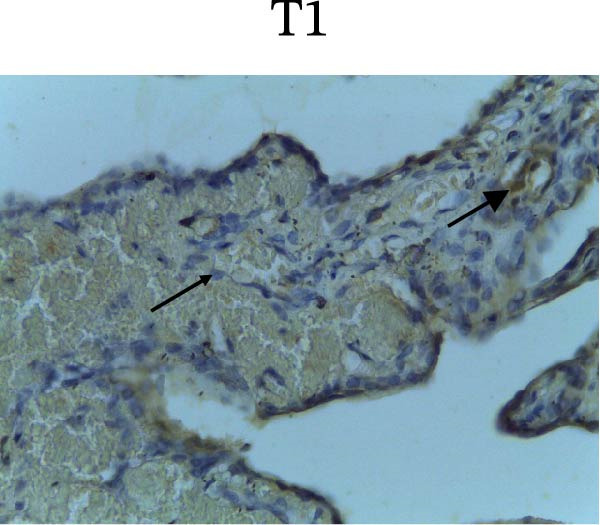
(H)

**Figure:**
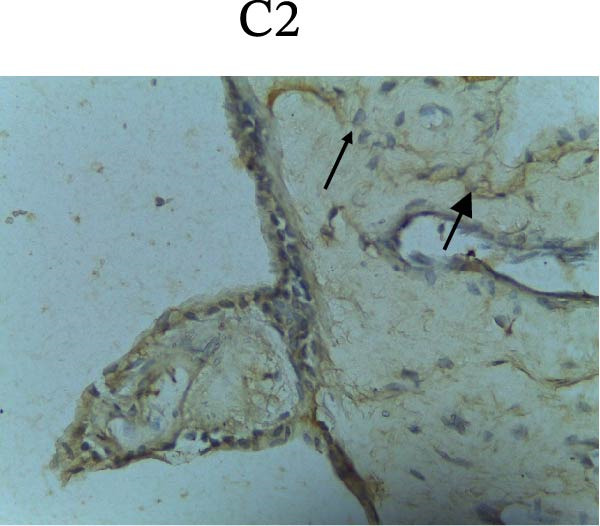
(I)

**Figure:**
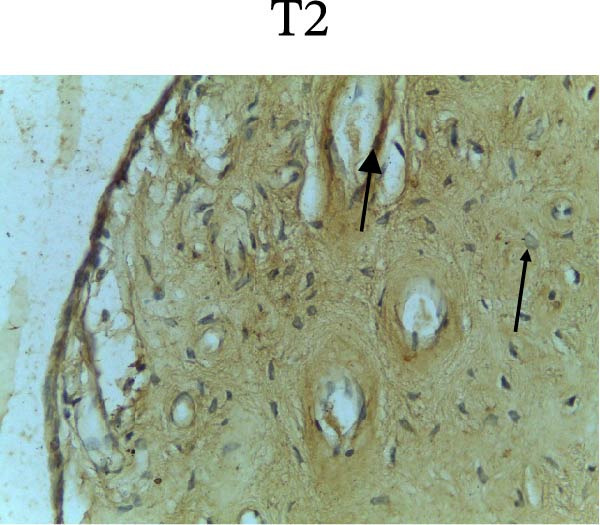
(J)

**Figure:**
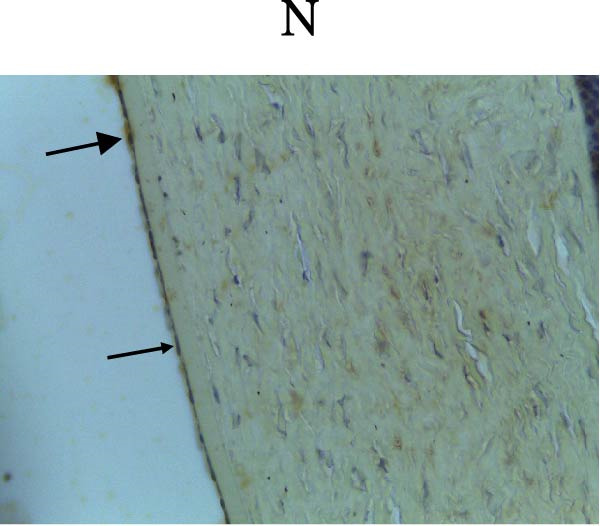
(K)

**Figure:**
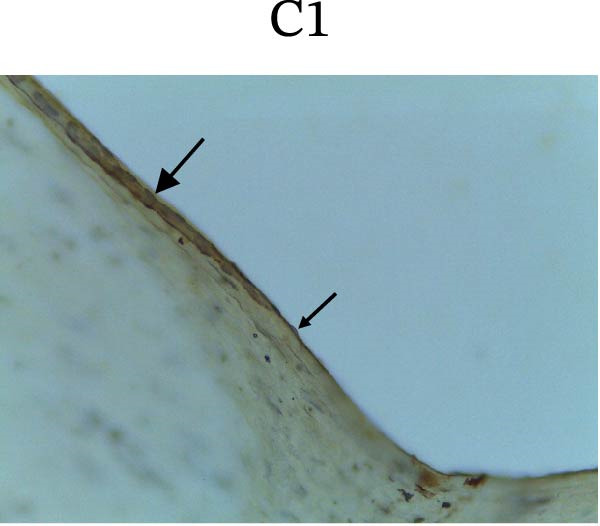
(L)

**Figure:**
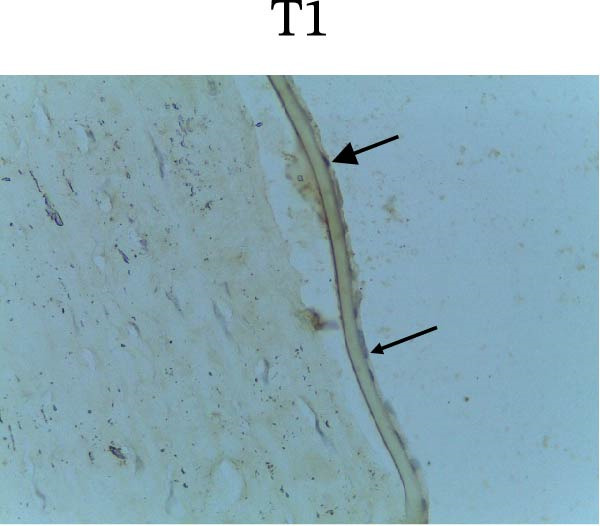
(M)

**Figure:**
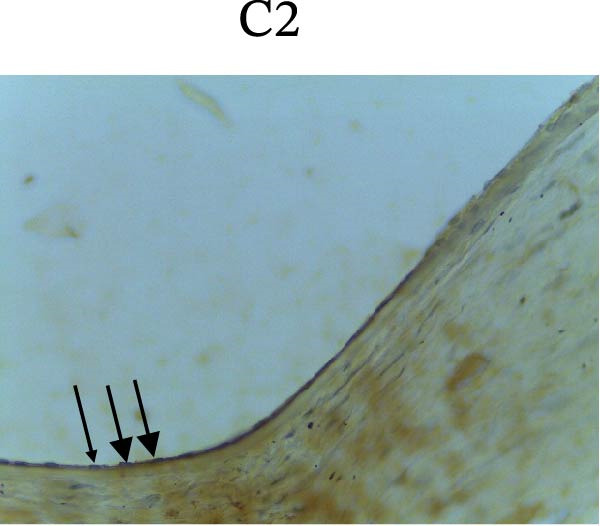
(N)

**Figure:**
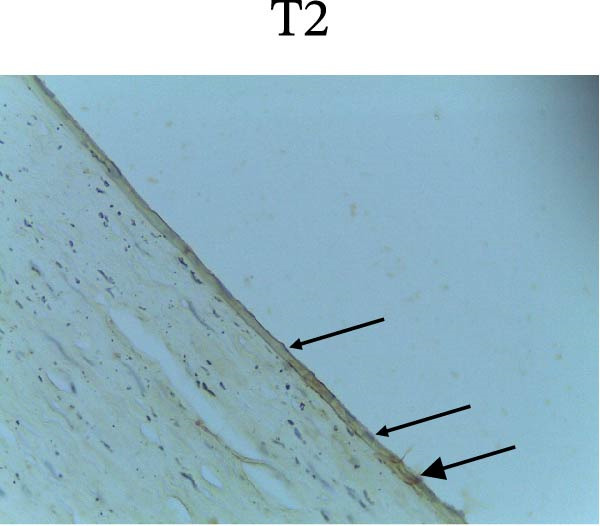
(O)

### 3.2. Results of Statistical Calculations for Comparisons Between Groups

IHC results for NF‐κB, TNF‐α, and Caspase‐8 expression showed normal distribution for NF‐κB expression, whereas TNF‐α and Caspase‐8 expression were not normally distributed. In the test of homogeneity of variances for NF‐κB expression, the results were not homogeneous (*p*  < 0.001). The difference test between groups for NF‐κB expression used the Brown–Forsythe test with the result that there was a significant difference (*p*  < 0.001), so it was continued with the Games–Howell test (Figure 3). The difference test between groups for TNF‐α and Caspase 8 expression used the Kruskal–Wallis test with the statistical test results that there was a significant difference in both cytokines (*p*  < 0.001), so it was continued with the Mann–Whitney test (Figure 3).

Figure 3Results of the intergroup difference test for each cytokine. (A) NF‐κB with Games–Howell test. (B) TNF‐α and (C) Caspase‐8 with Mann–Whitney test. Significant at *α* = 0.05.

**Figure:**
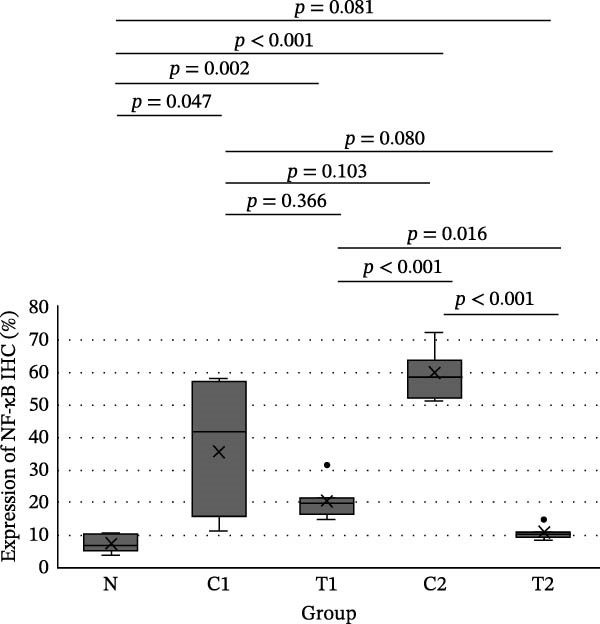
(A)

**Figure:**
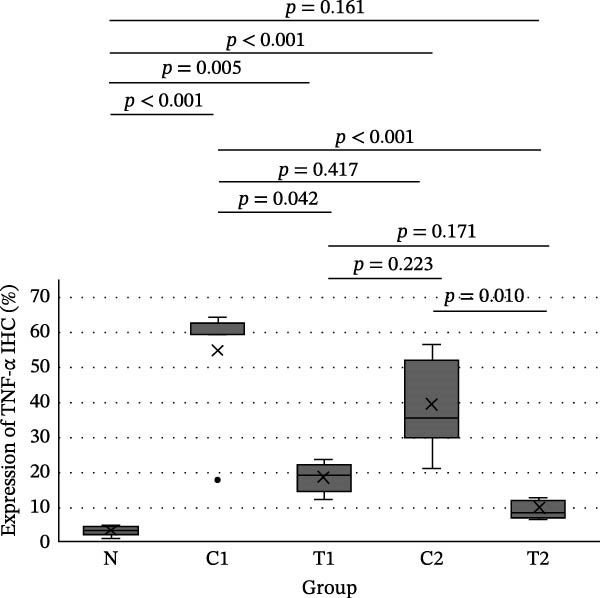
(B)

**Figure:**
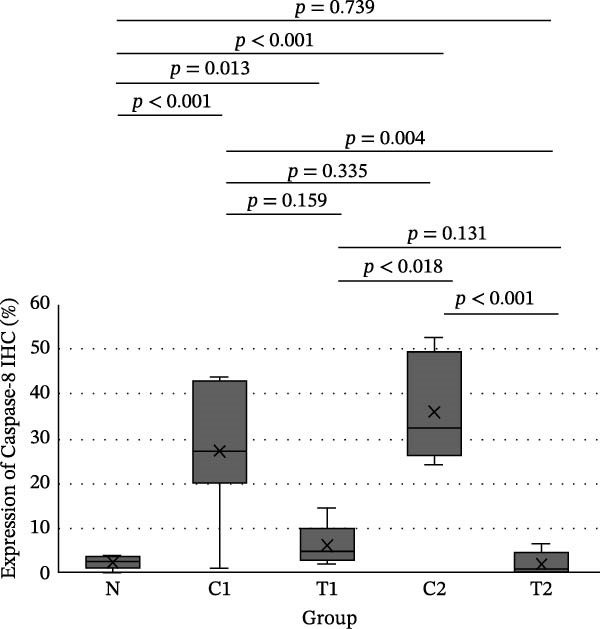
(C)

In Figure 3, for all inflammatory cytokines studied in this research, there is a decrease in expression in the group treated with LMSC‐S compared to the control. In the group treated with LMSC‐S, the cytokine expression closest to the N group was the T2 group.

### 3.3. Cytokine Expression Results Approaching the Normal Group (N)

Table 1 shows the average NF‐κB expression in macrophage cells for each group, from lowest to highest: N, T2, T1, C1, and C2.

**Table 1:** The average NF‐κB expression of macrophage cells.

Group	*N*	Mean ± SD (%)	Median	Min	Max	*p* (Brown–Forsythe)
N	8	7.36 ± 2.55^a^	6.70	4.10	10.80	<0.001
C1	7	35.67 ± 19.75^cd^	41.80	11.50	58.20	
C2	7	59.19 ± 7.59^d^	58.30	51.20	72.20	
T1	7	20.40 ± 5.44^bc^	19.80	14.90	31.60	
T2	7	10.79 ± 2.06^a^	10.40	8.30	14.90	

*Note*: Significant at *α* = 0.05. The same superscript letters show no difference between groups (Games–Howell).

Table 2 shows the average TNF‐α expression in macrophages for each group. The average expression of TNF‐α in macrophage cells from lowest to highest is Groups N, T2, T1, C2, and C1.

**Table 2:** The average TNF‐α expression of macrophage cells.

Group	*N*	Mean ± SD (%)	Median	Min	Max	*p* (Kruskal–Wallis)
N	8	3.43 ± 1.37	3.80^a^	1.00	4.80	<0.001
C1	7	55.02 ± 16.52	60.10^d^	17.76	64.10	
C2	7	39.57 ± 12.86	35.70^cd^	20.90	56.60	
T1	7	18.64 ± 3.98	19.40^bc^	12.50	23.50	
T2	7	9.37 ± 2.54	8.40^ab^	6.20	12.70	

*Note*: Significant at *α* = 0.05. The same superecript letters show no difference between groups (Mann–Whitney).

Table 3 shows the average Caspase‐8 expression in CECs for each group. The average expression of Caspase‐8, from lowest to highest, is T2, N, T1, C1, and C2.

**Table 3:** The average Caspase‐8 expression of corneal endothelial cells.

Group	*N*	Mean ± SD (%)	Median	Min	Max	*p* (Kruskal–Wallis)
N	8	2.45 ± 143	2.55^a^	0.00	4.10	<0.001
C1	7	27.40 ± 14.62	27.40^bc^	0.90	43.90	
C2	7	35.80 ± 10.99	32.60^c^	24.20	52.60	
T1	7	6.30 ± 4.45	4.80^ab^	2.00	14.50	
T2	7	2.04 ± 2.51	1.00^a^	0.00	6.50	

*Note*: Significant at *α* = 0.05. The same superscript letters show no difference between groups (Mann–Whitney).

## 4. Discussion

In this study, exposure to U/S power resulted in a significant increase in NF‐κB, TNF‐α, and Caspase‐8 expression in groups C1 and C2 compared with group N. LMSC‐S injection in groups T1 and T2 reduced the expression of these pro‐inflammatory cytokines, and the treatment group with the closest cytokine expression to Group N was Group T2.

Pro‐inflammatory cytokine expression decreased significantly between the control and treatment groups, except for NF‐κB and Caspase‐8 expression between groups C1 and T1; these decreases were not statistically significant. This could be due to the small sample size, the reciprocal relationship between NF‐κB and Caspase‐8, which is necessary for the balance between cell survival and death in response to damage during the early stages of trauma, or because the presence of LMSC‐S in the anterior chamber is short‐lived, so administration during U/S power exposure may not provide the expected anti‐inflammatory effect.

The relationship between these cytokines is complex. NF‐κB can increase TNF‐α expression, which, in turn, can activate Caspase‐8, leading to apoptosis. Conversely, Caspase‐8 can also regulate NF‐κB activity, thereby affecting the balance between cell survival and death in response to damage [58, 59]. These interactions are critical in determining cell fate after injury. If NF‐κB activation is sustained, it can promote cell survival and inflammation, while excessive TNF‐α signaling and Caspase‐8 activation can lead to apoptosis and tissue damage [60, 61]. Therapy of intracameral injection of LMSC‐S decreased NF‐κB, TNF‐α, and Caspase‐8 expression. In theory, the use of U/S power in phacoemulsification can damage CECs. Damaged CECs are DAMPs that activate macrophages. DAMPs activate NF‐κB in macrophages. Activated NF‐κB translocates to the nuclei of macrophages and induces the synthesis of pro‐inflammatory cytokines, such as IL‐1, IL‐6, IL‐8, and TNF‐α [45, 46]. NF‐κB regulates TNF‐α expression, whereas the TNF‐α autocrine–paracrine loop activates the expression of the NF‐κB transcription factor [47]. In this condition, it is likely that the synthesized TNF‐α is captured mainly by the TNFR2 receptor, which plays a greater role in maintaining cell life; therefore, the decrease in Caspase‐8 expression may be due to less TNF‐α being captured by the TNFR1 receptor.

An earlier study by An et al. [62] and Buono et al. [63] can help explain the decrease in Caspase‐8 expression observed following MSC secretome injections. In the study by An et al. [62], administering conditioned media (CM) from BMMSC reduced mitochondrial membrane potential changes, thereby increasing the survival of corneal epithelial cells exposed to nitrogen mustard (NM). The study by An et al. [62] differs from ours, where An et al. [62] studied the damage to corneal epithelial cells due to exposure to NM by administering therapy from CM or secretome from BMMSC. However, the effect on the apoptotic process was similar to that of the T2 group in our study, in which the intracameral injection of LMSC‐S significantly decreased Caspase‐8 expression in CECs. Decreased Caspase‐8 expression reduces Bid cleavage (a pro‐apoptotic Bcl‐2 family protein), maintains mitochondrial permeability transition pore opening, and reduces the release of cytochrome c and other apoptogenic factors into the cytosol. Decreased release of several of these factors reduces Caspase‐9 activation, which, in turn, reduces Caspase‐3 activation and weakens the apoptosis signal [48]. In a Buono et al. [63] study, the administration of extracellular vesicles (EVs) from BMMSC reduced proapoptotic Caspase‐3 levels under stressful conditions. Buono et al. [63] examined the protective effects of EVs derived from BMMSC on endoplasmic reticulum stress‐mediated apoptosis of CECs, which play a crucial role in the development of corneal endothelial dystrophy. The results of Buono et al. [63] study align with our findings, where EVs, a component of the MSC secretome (including limbus and bone marrow), can reduce the apoptosis process [19, 64].

Several factors should be considered when administering intracameral LMSC‐S therapy, particularly the apoptotic process following postphacoemulsification damage to the corneal endothelium. The decrease in apoptosis is not expected to lead to uncontrolled cell growth, unlike in cancer cases. The proliferation, invasion, and metastasis of tumor cells can be triggered by increased levels of pro‐inflammatory cytokines, such as IL‐1, IL‐6, and TNF‐α, which are induced by various factors, including obesity [65]. The secretome, a collection of factors or molecules secreted into the extracellular space by MSCs, has the advantage of reducing the risk of tumor formation due to its cell‐free composition [19]. However, it is still necessary to be aware of its potential for tumor growth through uncontrolled cell proliferation. Tumor cells are associated with obesity due to impaired hemostasis, caused by increased secretion of adipocytokines, pro‐inflammatory cytokines, growth factors, and hormones. This results in a chronic inflammatory process that leads to the accumulation of immune cells, including neutrophils, mast cells, T and B lymphocytes, and NK cells [65]. In our study, the inflammatory reaction that occurred was an acute inflammatory response triggered by innate immunity. CEC damage after U/S power exposure triggers innate immunity through an initial response to DAMPs captured by macrophages, followed by NF‐κB activation, which leads to the production of pro‐inflammatory cytokines, such as TNF‐α. TNF‐α will be captured by macrophages and CECs, leading to apoptosis via Caspase‐8 [46, 66, 67]. The role of LMSC‐S, as shown in our research, is to suppress inflammatory responses and apoptosis by decreasing NF‐κB expression, which in turn reduces TNF‐α and Caspase‐8 expression. The potential for uncontrolled cell growth leading to tumors or cancer from LMSC‐S therapy must be considered in terms of the expression of Ki‐67, CD166, HSP‐90, and p53 in CECs. Decreased HSP‐90 expression, along with increased expression of p53, Ki67, and CD‐166, indicates cell growth that can lead to tumors or cancer. HSP‐90 and p53 play crucial roles in regulating the cell cycle and apoptosis. HSP‐90 assists in the proper folding and function of p53 [68–72].

The anterior chamber of the eye is filled with aqueous humor secreted by the nonpigmented ciliary epithelium, produced at a rate of 2–3 µL/min, which decreases to 1 µL/min at night [73]. The volume of aqueous humor in the anterior chamber is 0.25–3.0 mL in rabbits and 0.24–0.28 mL in humans [74]. The anterior chamber has immune privilege (anterior chamber‐associated immune deviation, immunosuppressive factors in the aqueous humor, and FasL) [75]. Specific locations in the body (the brain, anterior chamber, and testes) are referred to as immunologically privileged sites because antigens within them do not trigger an immune response. In general, privileged sites are protected by a blood‐tissue barrier with low permeability to hydrophilic compounds. Functionally insignificant complement levels reduce the threat of acute inflammatory reactions and unusually high concentrations of immunosuppressive cytokines, such as IL‐10 and TGF‐β [45]. The secretome of LMSC contains IL‐10 and TGF‐β, at an equivalent level to that of the secretome of MSCs from bone marrow and adipose tissue [57].

The inflammatory phase begins after hemostasis is achieved. Neutrophils are the dominant cell type present within 24–36 h after injury. Guided by chemokines and other chemotactic agents, neutrophils migrate from the bloodstream to the wound environment through a process known as margination and diapedesis. Monocytes (macrophage precursors) appear in the wound (48–72 h after injury) and continue the process of phagocytosis. The proliferative phase begins on the third day after the initial attack and lasts for approximately 2 weeks. This is characterized by fibroblast migration, the deposition of newly synthesized extracellular matrix, and the formation of abundant granulation tissue [76]. In the wound healing process, the inflammatory phase begins 24–36 h after injury. The administration of LMSC‐S during U/S power exposure is likely to have been replaced by aqueous humor (production rate 2–3 µL/min) within 2.5 h, so the anti‐inflammatory effect of MSC secretome is not optimal. LMSC‐S was administered 3 days after U/S exposure, while monocytes (macrophage precursors) appeared 48–72 h after exposure. The anti‐inflammatory cytokine content in the secretome seems to help suppress macrophage activity during this inflammatory phase.

## 5. Conclusion

This study proposes that the intracameral injection of LMSC‐S on the third day after U/S power exposure may offer a novel strategy to prevent CEC loss by reducing apoptosis. The weakness of this study is the assessment of Caspase‐8 expression in the form of a percentage of CECs with positive expression compared to the total number of cells observed, which is a picture of a decrease in the apoptosis process or regeneration ability of rabbit CECs, considering that rabbit CECs still have regeneration ability. In contrast, human CECs do not have regeneration ability; these results still need to be seen if they are applied to the human eye.

## Author Contributions

**Dicky Hermawan**: conceptualization, data curation, formal analysis, funding acquisition, investigation, methodology, project administration, resources, software, validation, visualization, writing – original draft, writing – review and editing. **I. Ketut Sudiana**: conceptualization, formal analysis, methodology, supervision. **Fedik Abdul Rantam**: conceptualization, formal analysis, methodology, supervision. **Evelyn Komaratih**: conceptualization, formal analysis, methodology, supervision.

## Funding

The authors did not receive any financial support from public or private sources. There are no scholarships or fees from other parties.

## Ethics Statement

The research ethics was approved by the Animal Care and Use Committee (ACUC) of the Faculty of Veterinary Medicine, Universitas Airlangga, under Code of Ethics Number 2 KEH 122.09.2022 dated September 13, 2022. All applicable international, national, and/or institutional guidelines for the care and use of animals are followed. All procedures carried out in research involving animals complied with the ethical standards of the Faculty of Veterinary Medicine, Universitas Airlangga, Surabaya, Indonesia. This research was reported following the guidelines of ARRIVE (https://arriveguidelines.org). The research was conducted following the Guide for the Care and Use of Laboratory Animals (NIH Publication Number 85‐23, revised 1996).

## Conflicts of Interest

The authors declare no conflicts of interest.

## Data Availability

Research data supporting this publication are available from Google Drive at https://drive.google.com/drive/folders/1UsSF_98fu197NFZLRcDLVZm-Xyhg3seS?usp=sharing.
